# Supplementary material for: Synthesis and Characterization of Polyaniline/Carbon Nanodots: Electrochemical Sensing of Alcohols for Freshness Monitoring for Application as Packaging Materials
Source: Nanomaterials (Basel). 2025 Apr 12;15(8):593. doi: 10.3390/nano15080593 (PMC12029245; doi:10.3390/nano15080593)
Supplement: Supplementary file 1 [file nanomaterials-15-00593-s001.zip › nanomaterials-3556427-supplementary.pdf]

Supporting information

on

Synthesis and Characterization of Polyaniline/Carbon Nanodots: Electrochemical Sensing of Alcohols for Freshness Monitoring for Application as Packaging Materials

Shaila Jackson [1], Mary Taylor [1], Rajeev Kumar [1], Amit Kumar Shringi [1], TinChung Leung [2,3] and Ufana Riaz [1,2,*]

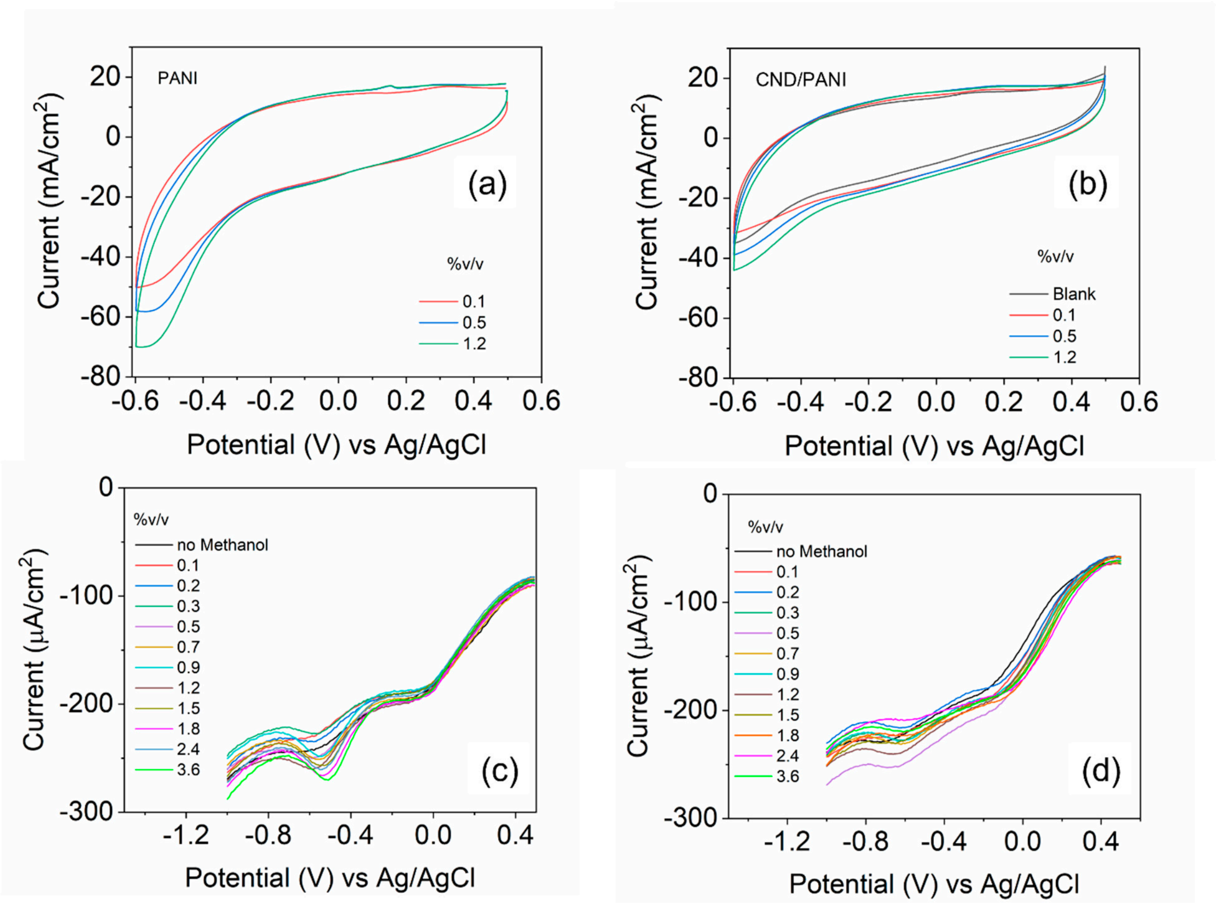

Figure S1. Cyclic voltammograms of (a) PANI, and (b) 2-CND/PANI using methanol as an analyte.
